# Effectiveness of a 16-Week Multimodal Exercise Program on Individuals With Dementia: Study Protocol for a Multicenter Randomized Controlled Trial

**DOI:** 10.2196/resprot.6792

**Published:** 2017-03-03

**Authors:** Sandra Trautwein, Andrea Scharpf, Bettina Barisch-Fritz, Christina Niermann, Alexander Woll

**Affiliations:** ^1^ Institute of Sports and Sports Science Karlsruhe Institute of Technology Karlsruhe Germany

**Keywords:** physical activity, dementia, postural balance, gait, activities of daily living, cognition, exercise

## Abstract

**Background:**

The increasing prevalence of dementia in the next decades is accompanied by various societal and economic problems. Previous studies have suggested that physical activity positively affects motor and cognitive skills in individuals with dementia (IWD). However, there is insufficient evidence probably related to several methodological limitations. Moreover, to date adequate physical activity interventions specifically developed for IWD are lacking.

**Objective:**

This study aims to investigate the effectiveness of a multimodal exercise program (MEP) on motor and cognitive skills in IWD in a high-quality multicenter trial.

**Methods:**

A multicenter randomized controlled trial with baseline and postassessments will be performed. It is planned to enroll 405 participants with dementia of mild to moderate stage, aged 65 years and older. The intervention group will participate in a 16-week ritualized MEP especially developed for IWD. The effectiveness of the MEP on the primary outcomes balance, mobility, and gait will be examined using a comprehensive test battery. Secondary outcomes are strength and function of lower limbs, activities of daily living, and cognition (overall cognition, language, processing speed, learning and memory, and visual spatial cognition).

**Results:**

Enrollment for the study started in May 2015. It is planned to complete postassessments by the beginning of 2017. Results are expected to be available in the first half of 2017.

**Conclusions:**

This study will contribute to enhancing evidence for the effects of physical activity on motor and cognitive skills in IWD. Compared to previous studies, this study is characterized by a dementia-specific intervention based on scientific knowledge, a combination of motor and cognitive tasks in the intervention, and high standards regarding methodology. Findings are highly relevant to influence the multiple motor and cognitive impairments of IWD who are often participating in limited physical activity.

**Trial Registration:**

German Clinical Trials Register DRKS00010538; https://drks-neu.uniklinik-freiburg.de/drks_web/navigate.do?navigationId=trial.HTML&TRIAL_ID=DRKS00010538 (Archived by WebCite at http://www.webcitation.org/6oVGMbbMD)

## Introduction

Dementia is one of the most frequently occurring diseases in the elderly [1], and the World Health Organization has declared dementia a public health priority [2]. The current prevalence of dementia is estimated at 47 million worldwide [3] and will presumably increase because of expected demographic changes [4]. This increasing prevalence (expected 135 million in 2050 [5]) will be accompanied by several societal and economic problems including rising disease-related costs and increasing demands for caregiving [2].

Dementia is a syndrome which comprises several different types of usually chronic and progressive diseases of the brain (eg, Alzheimer disease or vascular dementia) [6]. It encompasses diverse impairments and symptoms which affect individuals with dementia (IWD) in different ways depending on dementia type [7]. According to the *International Statistical Classification of Diseases and Related Health Problems, Tenth Edition* (ICD-10) [6], a diagnosis of dementia minimally requires the following symptoms: an impaired memory, further cognitive disturbances, and noncognitive disorders such as disturbed emotional control. These impairments potentially influence activities of daily living (ADL) [6] accompanied by an increasing loss of independence to a greater or lesser extent [8]. In addition, IWD suffer from motor and functional impairments such as affected gait and balance performance as well as transfer movements, which are not only reported in advanced stages [9-11].

To date, there is no cure for dementia, and commonly used medications for treating the symptoms of dementia have side effects emphasizing the urgent need for nonpharmacological interventions [12]. For instance, there is evidence that physical activity positively affects motor and cognitive skills of cognitively healthy elderly people [13]. Moreover, the number of studies analyzing this issue in IWD has increased [14-18]. For this sample, there are also systematic reviews and meta-analyses examining the effects of physical activity on balance, mobility, and gait as well as strength and ADL. Regarding balance, 3 of 5 reviews reported no or no clear benefit of physical activity [19-21] with largely varying effect sizes from small negative to large positive values [22,23]. Even if a positive effect of physical activity on mobility can be reported [19,21,24], the overall conclusion is inconsistent [20] with effect sizes ranging from small negative to large positive values [22,23]. Only a few reviews have considered specific aspects of gait function. One review has shown no to medium effect sizes for normal gait speed [22]. Reviews focusing on strength of lower limbs and ADL mainly reported improvements [19-26]. However, the small number of high-quality studies and the large heterogeneity in methods used in these studies represent insufficient evidence regarding the effects of physical activity [22,26].

Reviews and meta-analyses examining the effects of physical activity on cognitive skills in IWD mainly assess overall cognition. Of 6 reviews and meta-analyses, 3 found no evidence for the benefit of physical activity on cognition in IWD [20,26,27] while the others found a positive overall effect [12,19,28]. Groot et al [12] stated that overall effects of physical activity on cognition are comparable to the effect size observed in meta-analyses examining the effectiveness of pharmacotherapy in IWD [29-31].

Most of the systematic reviews and meta-analyses suggest even if evidence is lacking that physical activity positively affects IWD, for example, in balance, mobility, and cognition. Their conclusions are that there is an urgent need for high-quality intervention studies [12,20,22,23,27]. In their opinion, methodological shortcomings including insufficient reporting of methods and results and small samples as well as the use of inadequate outcome measures [12,22,27,28] could be responsible for the lack of conclusive evidence. Furthermore, Hauer et al [32] discussed that low effectiveness of existing physical activity interventions may explain negative or inconsistent findings in previous studies. It can be speculated that the effectiveness of existing training interventions is limited by inappropriate intensity, duration, type of training, lack of specific interventions, or individualization of training [32].

This study will investigate the effects of a physical activity intervention on motor and cognitive skills. The intervention focuses on dementia-specific motor deficits and aims to influence the underlying motor performance, which depends on complex cognitive processes like integrating sensory information, central processing, or efferent motor output [33]. This reflects the close connection between cognitive and motor functions and could provide insights in disease progression [34]. It is highly relevant for IWD to counteract and possibly reduce dementia-related motor deficits which typically result in distinct constraints of mobility-dependent quality of life as well as loss of independence and higher risk for falls [35-37]. Hence, primary outcomes are based on 3 considerations: dementia-specific motor deficits, relevance for everyday life, and measurement quality (direct and feasible measurements). Balance, gait, and mobility fulfill all requirements and influence quality of life [9,10,38,39]. ADL are defined as secondary outcomes because they are considered an entire construct related to several motor and cognitive skills. Thus, measuring ADL is more difficult and less objective than measuring balance, mobility, and gait. Further, we chose strength and function of lower limbs and cognition as secondary outcomes because of their expected influence on primary outcomes.

Aiming to overcome the above mentioned methodological limitations, we will realize a high-quality multicenter trial with a sustainable intervention close to everyday life. The following aims will be addressed.

Primary aim: to determine the effect of a multimodal exercise program (MEP) compared to conventional treatment (eg, medication, care, therapeutic applications) on balance, mobility, and gait. We hypothesize that a 16-week MEP in addition to conventional treatment affects balance, mobility, and gait in IWD more than the conventional treatment. Additionally, we will compare different subgroups (eg, according to sex, stage of dementia, or attendance).

Secondary aim: to investigate the influence of mediator and moderator variables on primary outcome measures. We assume that the effects of physical activity on balance, mobility, and gait are caused or influenced by changes in underlying motor and cognitive skills.

Comparably, we will investigate the effect of MEP on the secondary outcomes strength and function of lower limbs, ADL, and cognition as well as the effect of mediator and moderator variables on ADL. By addressing these aims, this study contributes to enhancing evidence concerning the effects of physical activity on motor and cognitive skills in IWD.

## Methods

### Study Design

The study design has been primarily defined to address the primary aim of the study on the effectiveness of a 16-week MEP. For this reason, we will perform a multicenter randomized controlled trial with baseline and postassessments and an allocation ratio of 2:1 for intervention (IG) and control group (CG), respectively. Ethical approval has been obtained from the ethics commission of the Karlsruhe Institute of Technology. The study is retrospectively registered in the German National Register of Clinical Trials [DRKS00010538]. This study protocol considers guidelines and recommendations of the Standard Protocol Items: Recommendations for Interventional Trials (SPIRIT) [40,41] and Consolidated Standards of Reporting Trials (CONSORT) statements [42-44].

### Participants

Participants for this study will be recruited in public, private, and charitable care facilities in southwestern Germany, in particular in the metropolitan region Rhein-Neckar and the district around Karlsruhe. All randomly selected care facilities offer inpatient care for approximately 60 to 300 residents and provide a common room where the intervention will be performed. A total of 3 recruitment periods with consecutive sampling within each care facility are planned.

Employees of care facilities will identify possible participants with the purpose to fulfill selection criteria.

Inclusion criteria include (1) diagnosis of dementia or suspected dementia (based on the assessment of the objective ICD-10 criteria by employees and the examination of cognitive abilities with Mini-Mental State Examination [MMSE][45]), (2) Alzheimer disease, vascular dementia, or other primary dementia (all types caused by neurodegenerative or vascular diseases: eg, lewy body dementia or frontotemporal dementia [46]), (3) mild to moderate stage of dementia (MMSE 10-24), (4) age above 65 years, (5) walking ability of approximately 10 meters with or without walking aid, and (6) clearance by general practitioner.

Exclusion criteria include (1) secondary dementia (all types resulting from organic illness or injury: eg, toxic substances or brain injuries [46]), (2) other severe cognitive impairments, (3) other severe neurological disease, (4) other severely acute diseases, and (5) severe motor impairments.

**Figure 1:**
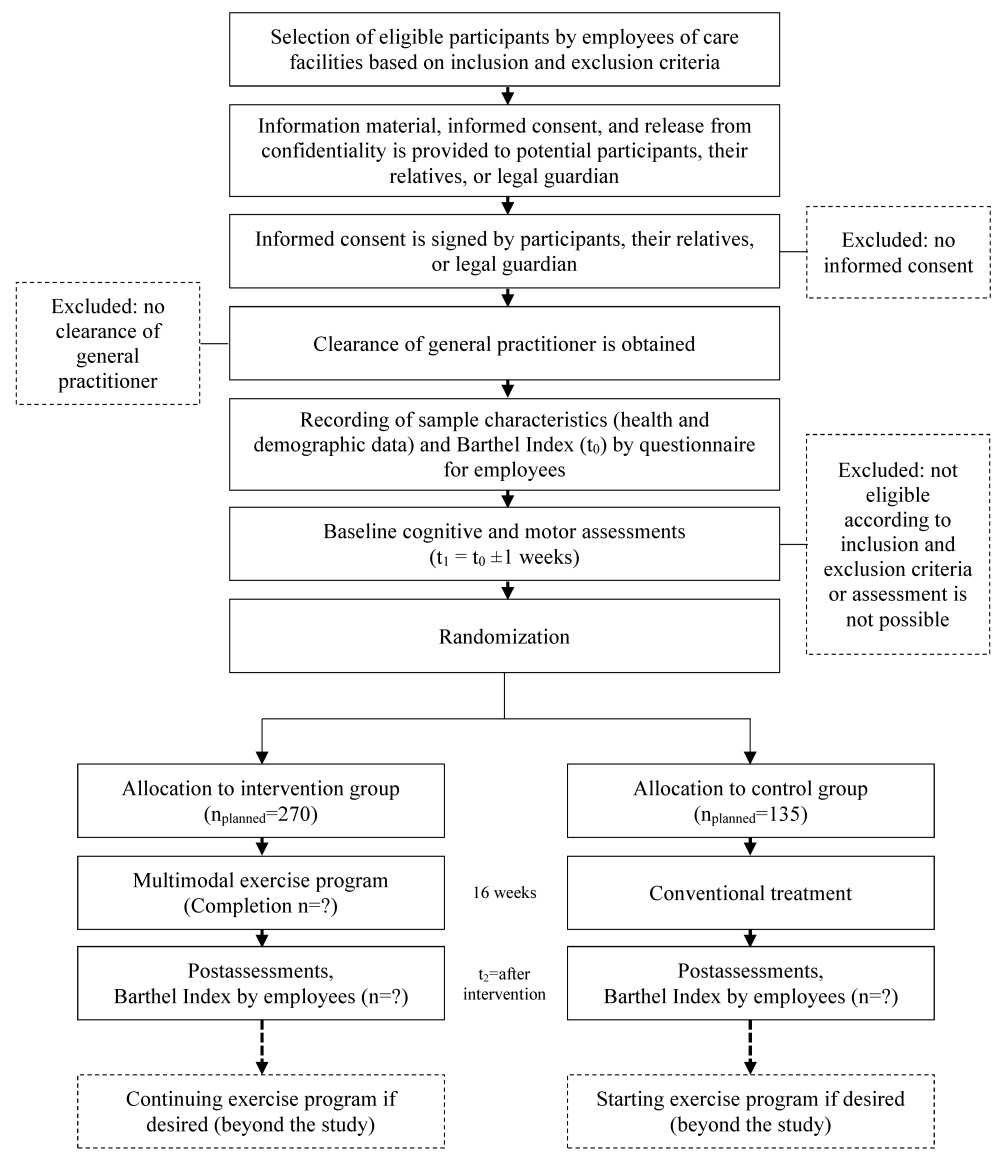
Flow of participants.

Potential participants will receive a comprehensive information letter and an informed consent form, which will be signed by individuals or their legal guardians prior to the study. The informed consent along with clearance of participant’s general practitioner allow scheduling of baseline assessments where eligibility will be verified according to the inclusion and exclusion criteria. Flow of participants is illustrated in Figure 1.

### Intervention

The intervention is specifically developed for this study based on theoretical considerations, results of a pilot study [47], and a literature review [48]. The combination of motor and cognitive tasks used in the MEP aims to enhance the effectiveness of physical activity on cognition. This is theoretically supported by findings in healthy older adults showing that the combination of both yields larger effects on cognition than using each alone [49]. The pilot study (n=19) aimed to prove feasibility of the intervention and allowed first insights regarding the effectiveness. After a 10-week intervention, IG showed no significant changes in Alzheimer Disease Assessment Scale–Cognitive Subscale (ADAS-Cog, German version) [50] sum score but significant improvements in subscore orientation/praxis. In contrast, we found a significant decline in ADAS-Cog sum score of CG [47]. Moreover, IG showed significant improvements in get-up-and-go test whereas CG did not significantly improve (unpublished results). The literature review aimed at giving recommendations for designing interventions for IWD. Analyzed studies showed that a physical activity intervention for IWD should at least last 4 months with 2 to 3 sessions of 45 to 60 minutes per week. Moreover, interventions focusing on several motor skills (eg, endurance, strength, balance) seemed to be more effective than interventions with only 1 task [48]. Hence, the 10-week intervention of the pilot study has been revised for the current study. The revision, which aimed to provide a balanced MEP with specific, adequate, and intensity-demanding tasks, comprises adjustments of contents (motor qualities as well as connection between motor and cognitive tasks) and intervention duration (extension to 16 weeks).

The MEP will be guided by 2 skilled instructors with experience in sports science and performed as group training mainly in a seated position. A group will consist of a maximum of 12 participants and will be joined by familiar caregivers to support the instructors if needed. The underlying didactic concept focuses on specific needs and characteristics of IWD and includes increased supervision realized by 2 instructors, adaptation to the cognitive level of participants, adjusted communication (eg, simple language, nonverbal aspects), ritualization to give orientation and familiarity, and adequate complexity by simple and well-structured cognitive and motor tasks.

To ensure high standards and comparability, each session is planned in detail and all instructors participate in a special training focusing on structure and contents of MEP as well as special demands resulting from the characteristics of IWD. A detailed training manual is provided for instructors, and the adherence to this manual will be emphasized. To ensure standardization, all tasks are described precisely and photographs are provided.

Providing a sense of security is an important aspect realized by ritualization. To satisfy this ritualization, the general sequence is identical for all sessions including an imagination of experienced journeys. Each session is divided into 3 parts: arrival, destination, and departure. Whereas arrival and departure remain consistent over the whole intervention period, a new travel destination is selected every time. A total sample session of MEP is found in Multimedia Appendix 1.

The arrival as beginning ritual of each training session takes about 5 to 7 minutes and aims to prepare participants for the following main part. Tasks for mobilization and stimulation of the cardiovascular system are linked to cognitive activation.

**Figure 2:**
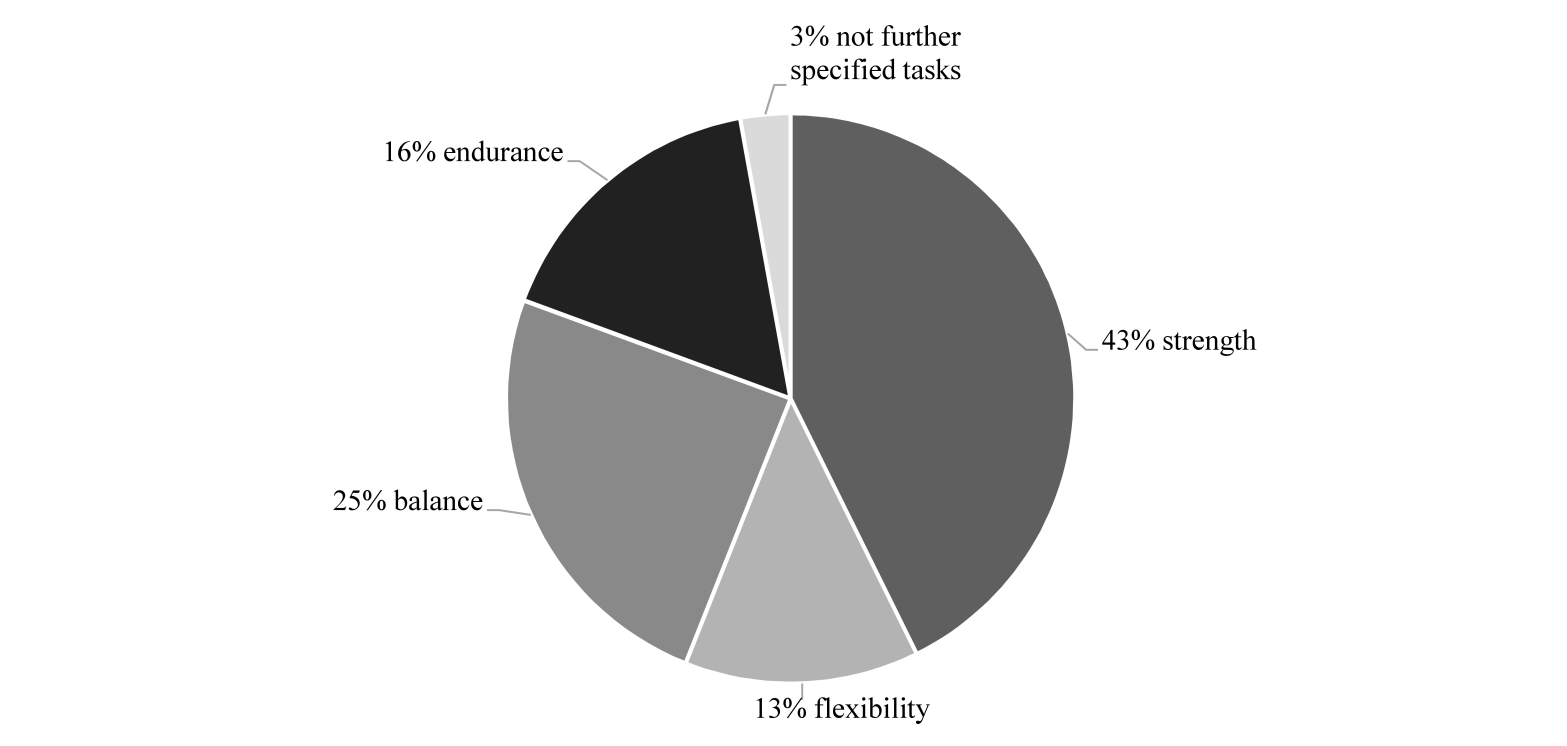
Distribution of motor qualities within the main parts of the multimodal exercise program.

The main part of MEP is the destination (about 35 minutes) which includes tasks for strength (43%), balance (25%), endurance (16%), flexibility (13%), and not further specified tasks (3%) (see Figure 2). In addition, cognitive tasks are incorporated to stimulate memory, attention, language, and executive functions. Tasks are carried out with medium to submaximal intensity. Throughout the intervention, there will be a progression concerning intensity as well as motor and cognitive requirements. Examples of different motor and cognitive tasks as well as examples for their progression are given in Table 1.

**Table 1:** Examples of motor and cognitive tasks of the multimodal exercise program and their progression.

		Simple performance	Progressive performance developed within the 16 weeks
**Strength**			
	Imagination/journey	Mediterranean cruise – aquafitness on the deck of the ship	Circus – task after tightrope dance
	Starting position	Seated, arms stretched above head	Standing upright behind chair, arms stretched above head
	Motor task	Lateral flexion with pool noodle	Lateral flexion with rope
	Sets and repetitions	3 sets with 2 repetitions for each side	2 sets with 3 repetitions for each side
	Muscle activity	Upper limbs and core	Upper limbs, core, and lower limbs
	Cognitive task	No additional cognitive task	Answering questions about circus performances (eg, Have you ever been to a circus? If yes: Which was the best circus act? If no: What do you think would be the most interesting thing if you visited a circus?)
**Balance**			
	Imagination/journey	Safari in Namibia – washing an elephant	World trip – washing an elephant
	Starting position	Seated, 1 arm is horizontally stretched, flexion in hip joint to shift body weight forward	Standing upright behind chair, one arm is horizontally stretched, flexion in hip joint to shift body weight forward
	Motor task	Slow and large arm movements in horizontal plane holding a small sandbag while leaning to left and right sides	Slow and large arm movements in horizontal plane holding a small sandbag while leaning to left and right sides
	Cognitive task	Answering questions about elephants (eg, Have you ever seen an elephant? Are there different kinds of elephants? What are the differences?)	Counting to 180 in steps of 6 (change hands at 90)
	Duration/repetitions	1 minute/approximately 10 repetitions per side	Approximately 1:30 minutes/15 repetitions per side
**Endurance**			
	Imagination/journey	Soccer World Cup – walking to the soccer training	On a treasure island – walking downhill through the jungle
	Starting position	Seated	Standing upright behind chair
	Motor task	“Walking” in seated position – lifting legs with active use of arms	“Walking” on the spot – lifting legs with active use of arms (if possible)
	Duration	1 minute	3 minutes
	Cognitive task	Answering questions about soccer and its rules (eg, Who knows some soccer rules? Do you know how many referees there are during a game?)	Naming animals living in the jungle. If a participant repeats an animal he or she is asked to name another one
**Flexibility**			
	Imagination/journey	Safari in Namibia – wood chopping for a campfire	Olympic Games – laola wave of the audience
	Starting position	Seated	Standing upright behind chair
	Motor task	Extention and flexion of the trunk, bringing arms in extention with maximal personal range of motion	Extention and flexion of the trunk, bringing arms in extention with maximal personal range of motion (try to increase range of motion)
	Set and repetitions/duration	3 sets with 10 repetitions (5 repetitions slow, 5 repetitions fast)	No repetitions defined, duration 3 minutes
	Cognitive task (example)	Performing in the same rhythm synchronous with other participants, 5 slow hits, 5 faster hits	Learning 3 different signals: 1= moving fast, 2= moving slow, 3= change direction of laola wave, performing according to signals

The departure takes about 5 minutes and aims to cool down and relax the body while leading participants out of imagination and back into reality. Similarly to the arrival, instructors guide participants through fixed sequences.

The MEP takes place twice a week on nonconsecutive days over a period of 16 weeks. Each session lasts 60 minutes with motor and cognitive tasks taking about 45 minutes to ensure sufficient time for rests and explanations. Prior to the first session, a social gathering session is held aiming for an initial familiarization and information acquisition with regard to participants and care facilities. Attendance and adherence of participants will be documented by instructors for each session. Adherence will be assessed using a short formula to rate attention, participation, motivation, and behavior of each participant.

Conventional treatment comprising, for instance, medication, care, or therapeutic applications is individually tailored and will be continued in all included participants of CG as well as IG.

### Outcomes

#### Determination of Outcomes

Primary outcomes refer to the motor qualities balance, mobility, and gait. Secondary outcomes are other motor variables such as strength and function of lower limbs and ADL as well as cognitive variables assessing overall cognition, language, processing speed, learning and memory, and visual spatial cognition. All outcome parameters are listed in Table 2. The aim of this study is to investigate changes in outcomes between IG and CG. Furthermore, the focus is on differences in all outcome variables between baseline and 16-week postassessment.

**Table 2:** Primary and secondary outcome parameters.

	Outcome	Assessments (at baseline and 16-week postassessment)
**Primary outcomes**		
	Balance	Frailty and Injuries: Cooperative Studies of Intervention Techniques 4 (FICSIT-4) [51]
	Mobility	
		Timed Up and Go test [52]
		6-meter walk test [53]
	Gait	Gait analysis using GAITRite: temporal and spatial gait parameters (gait speed, cadence, cycle time, step length, step width, gait variability, single support, and double support)
		- Walking with normal speed
		- Walking with normal speed and the task counting backwards from 50
		- Walking with normal speed and the task naming animals
**Secondary outcomes**		
	Lower limb strength	Modified 30-second chair-stand test [54,55]
	Lower limb function	Short physical performance battery [56]
	Activities of daily living	
		Barthel Index (German version according to Hamburger Einstufungsmanual [57,58])
		Erlangen Test of Activities of Daily Living (E-ADL-Test) [59]
		7-item physical performance test [60]
	Overall cognition	Mini-Mental State Examination (MMSE) [45]
	Language	
		Verbal fluency “category animals”
		Phonemic fluency “S-words”
	Processing speed	Trail Making Test A [61,62]
	Learning and memory	
		California Verbal Learning Test, short version 1 [63]
		Digit span forward and backward [64]
	Visual spatial cognition	Clock drawing test [65]

The primary and secondary outcomes have been discussed in an international expert panel consisting of 14 scientists from 7 institutions in 3 countries (Germany, Australia, and Netherlands) with the disciplines sports science (especially focusing on locomotion research, sports therapy, kinesiology, biomechanics, training science, physical education and health, diagnostics, evaluation, and sports psychology), geriatrics/gerontology, psychology, and physiology. Among these experts, a standardized testing procedure has been determined focusing on relevance of outcomes as well as validity, reliability, objectivity, and feasibility of recording methods. The selected outcomes and recording methods are common in geriatric assessments and have been frequently used in previous studies examining IWD. However, it must be pointed out that most of recording methods regarding the motor qualities have not been developed for IWD. Feasibility of the test battery and recording procedure was tested in a sample of 20 participants prior to the current study. This pilot study proved feasibility of planned assessments in IWD.

Trained investigators with experience in sports science guide the baseline and postassessments in the care facilities. Prior to assessments, investigators participate in a special course to get detailed information about testing procedure and measurements. To standardize testing procedure and ensure comparability, a detailed testing manual is provided to which investigators are urged to strictly adhere. Accordingly, a detailed description of performing each assessment is given in Multimedia Appendix 2. Moreover, investigators will be educated about specific aspects of working with IWD.

#### Primary Outcomes

Static balance will be determined using the Frailty and Injuries: Cooperative Studies of Intervention Techniques 4 scale (FICSIT-4) [51]. Mobility will be assessed using the timed Up and Go test [52] and 6-meter walk test [53]. The 6-meter walk test aims to capture normal gait speed. To reduce bias caused by the testing situation, participants are not explicitly informed about time keeping.

Temporal and spatial gait parameters will be analyzed using the electronic gait analysis system GAITRite (CIR Systems Inc, Franklin, NJ) with an active length of 4.88 meters, a spatial resolution of 1.27 centimeters, and a scan rate of 120 hertz. The following parameters are of special interest: gait speed, cadence, cycle time, step length, step width, gait variability, single support, and double support (as percentage of cycle time). Gait parameters are recorded for 3 different conditions: walking with normal speed, walking with normal speed and the task of counting backwards from 50, and walking with normal speed and the task of naming animals.

**Figure 3:**
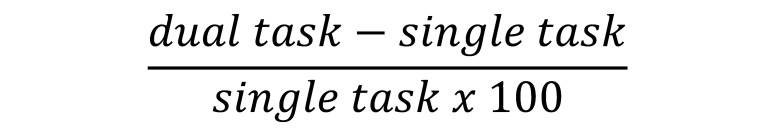
Calculation of changes in gait parameters caused by dual task.

Changes in gait parameters caused by dual task will be calculated using the equation seen in Figure 3. The generated value represents dual-task costs indicating the better performance under dual-task condition the lower this value is [66,67].

#### Secondary Outcomes

Strength of lower limbs will be determined by modified 30-second chair-stand test. In this modified version participants are allowed to use their arms [54,55], and the time to perform 5 repetitions is additionally measured. After a rest, fit participants complete a second trial without using arms with the same recording procedure as for the modified 30-second chair-stand test (including time for 5 repetitions). Function of lower limbs will be evaluated using the short physical performance battery, consisting of standing balance (Romberg, semitandem, tandem), gait speed, and 5 times sit-to-stand without using arms [56].

ADL will be determined using the Barthel Index (German version according to Hamburger Einstufungsmanual [57,58]), Erlangen Test of Activities of Daily Living (E-ADL-Test) [59], and 7-item physical performance test [60]. The Barthel Index will be completed by employees of the care facilities. To ensure standardized answers, employees receive a manual with detailed information. The E-ADL-Test and the 7-item physical performance test aim to practically examine ADL. Although the revalidation of the E-ADL-Test [59,68] showed that the tasks are too easy for mild dementia, for our target sample this test is considered as appropriate substantiated by the development for IWD. Furthermore, the E-ADL-Test is regarded as a valid and reliable instrument for assessing ADL of individuals with moderate to severe dementia [59,68].

Cognitive outcomes will be assessed using some subtests of the neuropsychological test battery Consortium to Establish a Registry for Alzheimer's Disease–Plus (CERAD-Plus) [69]. Overall cognition will be determined using MMSE [45]. Language will be examined regarding verbal fluency “category animals” and phonemic fluency “S-words.” The first fluency task provides information about verbal rate and fluency, semantic memory, language, executive function, and cognitive flexibility [70,71]. The second task examines fluency in a more strategic manner rather than the semantic memory. Processing speed and visual scanning will be determined using the Trail Making Test A [61,62]. In addition to CERAD-Plus, the California Verbal Learning Test, short version 1 (except forced choice recognition) [63], and digit span forward and backward [64] will be performed to assess learning and memory. Visual spatial cognition will be assessed using the clock drawing test [65].

Moreover, body mass and height will be measured using a Seca 813 Robusta scale and Seca 213 stadiometer (Seca, Hamburg, Germany) with an accuracy of 0.1 kilogram and 0.1 centimeter, respectively.

#### Sample Characteristics

Further possible influencing variables including age, medication, or other diseases are recorded chronologically close to baseline assessments. Employees of the care facilities will be asked to complete the health and demographic data questionnaire and the Cumulative Illness Rating Scale [72] for each participant. The questionnaire includes sex, year of birth, diagnosis of dementia, severity of dementia, type of dementia, date of diagnosis, depression, severity of depression, number of medications, medications for dementia, antidepressants, and walking aids. A written consent to collect these data by employees of the care facilities will be obtained from participants or their legal guardian.

### Sample Size

The required sample size was calculated via G*Power version 3.1.9.2 (Heinrich Heine University of Dusseldorf) [73], taking into account the following parameters: analysis of variance (ANOVA) for repeated measures, within-between interaction, small effect size (ɳ²=0.01, d=0.2) [74], 2-sided α-error of .05, power of .80 (1-β), and 2 groups and 2 measurements. The small effect size used for the calculation of required sample size is based on literature review and assumptions of relevant changes for IWD. Previous studies have reported high variation in the effect sizes of the primary outcomes balance, mobility, and gait. In their review, Blankevoort et al [22] reported small negative to large positive effect sizes for balance (d=–0.24 to d=3.59) and functional mobility (d=–0.25 to d=2.37) as well as no to medium effect sizes for normal gait speed (d=–0.11 to d=0.50). These reported variations do not allow determining actual effect sizes. Thus, the magnitude of relevant changes has to be considered to further support the selection of a small effect size. Because dementia is characterized by rapid progression linked to multiple impairments, it is assumed that even small effects are relevant. The calculation of sample size results in a required sample size of 100 participants for each group (total sample size of 200 participants). Considering reasons for dropout, the sample is set to 405 participants.

### Dropout

We assume 3 reasons for dropout: (1) withdrawal from the study, (2) missing data, and (3) low attendance or adherence to MEP. Possible reasons for withdrawal are death, hospitalization, serious deterioration in state of health, refusal to participate, etc. Based on the literature review of Blankevoort et al [22], a dropout rate of 20% caused by withdrawal is expected. Missing data occur if participants are not able to complete the entire test battery because of motivational aspects or multiple motor and cognitive impairments. In addition, some participants will not participate at all in postassessments because of illness or other appointments. We assume a missing data rate of 15%. A total target number of 200 participants (100 per group) for the analysis and an assumed dropout rate (withdrawal and missing data) of 35% requires enrolling 270 participants into the study. Unfortunately, attendance and adherence are often not stated in previous studies [26]. Hence, we decided to double the sample of IG to ensure the required sample of 100 participants in this group. Low attendance and adherence may be caused by illness, motivation, other appointments, disinterest, or other reasons. Hence, a total sample size of 405 participants is required.

All participants will be asked at least twice if they are willing to participate in the assessments to reduce missing data. A familiar caregiver is asked to invite the participant if appropriate. If participants are not willing to complete all measures they are offered to choose assessments they are willing to complete. Moreover, all possible participants will be included in the data collection regardless of whether they discontinued or deviate from the intervention protocol. Caregivers will be asked to support the participants to get to training sessions to improve attendance. If participants miss a session, they are personally invited to the next training session.

### Allocation

Group allocation to IG and CG will be performed by minimization to obtain randomized groups with minimum group differences. Subjects rather than care facilities will be randomized to avoid confounding effects of the geographic location, and minimization will be done separately for each care facility based on the baseline criteria MMSE, sex, age, and baseline performance of modified 30-second chair-stand test. Minimization will be performed with the program MinimPy version 0.3 [75], which includes a random element. The first participant is allocated randomly to IG or CG. Subsequent participants are allocated to each group correspondingly to achieve the least imbalance between groups. Including a random element, participants will be allocated to the better fitting group with a probability of 70%. An allocation ratio of 2:1 is selected because of above-mentioned assumptions regarding dropouts. The input order of participants for allocation will be randomly defined by an assigned number for each participant given prior to minimization.

### Blinding and Pseudonymization

Investigators will be blinded to allocation wherever possible. It is not possible to blind participants or employees of care facilities regarding group allocation.

All data is stored in a strictly pseudonymous form. This is achieved by separating personally identifiable information of participants from data collected during baseline and postassessments. Collation of data is only possible with considerable effort at any time of the study. Thus, individual confidentiality will be ensured before, during, and after the study. Only selected team members have access to coded data.

### Statistical Analysis

All statistical analysis will be done with SPSS version 23 (IBM Corp). Trained and experienced investigators will evaluate and enter data. Investigators evaluating and entering data are not the same as investigators assessing outcomes. The number of investigators is limited to 2 per assessment method. Prior to actual analysis, interrater reliability (Cohen kappa [76], intraclass correlation coefficient [77]) will be calculated and plausibility (eg, considering range and distribution) will be checked to minimize errors caused by data evaluation and entry.

Because of expected large dropout rate, which can lead to a critical amount of missing data, 2 separate analysis sets are planned: an intention-to-treat analysis and a per-protocol analysis. In the intention-to-treat analysis, all randomized participants regardless of protocol adherence will be included and missing data will be substituted by multiple imputation. Participants with sufficient attendance and adherence to the intervention as well as complete assessments of primary outcomes will be included in the per-protocol analysis, where missing data will not be considered.

Baseline values of participant characteristics will be compared between IG and CG using chi-square tests for categorical data, Mann-Whitney-U tests for nonparametric variables, and *t* tests for continuous and normally distributed parameters. For all normally distributed data (checked by Shapiro-Wilk test), mean and standard deviation will be calculated, and medians and interpercentile ranges will be calculated for not normally distributed data. Treatment effects will be analyzed using 2-factor ANOVA with repeated measurement. A 2-sided *P* value less or equal to .05 will be considered to indicate statistical significance. In addition, 95% confidence intervals and partial Eta² will be calculated. Changes in motor and cognitive function are possible mediators and moderators. These mediating and moderating effects on primary outcomes will be analyzed using multiple linear regression models. Additional explorative data analysis exceeding the proposed planned analyses will be performed. Depending on data structure, adequate analysis methods will be defined. These analyses aim to consider further influencing factors or subgroup analysis as well as the development of forecast models.

## Results

Enrollment for the study started in May 2015. It is planned to complete postassessments by the beginning of 2017. Results are expected to be available in the first half of 2017.

## Discussion

### Summary

Previous studies have discussed the use of physical activity as additional therapy strategy, and predominately positive effects have been reported. However, the results of these studies are not consistent and they have several methodological limitations. With respect to these limitations, the current study has been carefully designed and thus reflects the following strengths.

### Strengths

The overall strength is the strong effort to conduct a high-quality trial characterized by a standardized study design, theoretical considerations, an intervention specially designed for IWD, assessments adequate for IWD, a large sample size, and detailed and accurate reporting of methods according to the CONSORT [42-44] and SPIRIT [40,41] statements.

The MEP, which is characterized through dementia-specific methodology and a combination of motor and cognitive tasks, is a major strength of this study. Because of its theoretical foundation and based on primary recommendations of the review by Scharpf et al [48], initial guidelines for designing physical activity interventions for IWD can be derived if results support efficiency.

Bearing in mind that most motor assessments are not developed for IWD and their psychometric properties have hardly been systematically established in this specific population [55,78], we took several efforts to construct an adequate test battery considering all relevant primary and secondary outcomes. The international expert panel with members from different disciplines where we have discussed possible and adequate measurements as well as general information on performing cognitive and motor measurements in IWD has been an important attempt to enhance quality. In comparison to previous studies, the large sample size is an outstanding feature of this study. To the best of our knowledge, there is no other study with a comparable sample size. Based on studies analyzed for the Cochrane review [26], sample sizes vary between 12 and 148 participants.

This study is designed as a multicenter trial with a sustainable intervention close to everyday life. For instance, the MEP is established on everyday activities such as getting up, walking, or picking things up (see Multimedia Appendix 1). Performing a field study reflects reality in participating care facilities, and results can be more easily transferred to daily routine. Considering sustainability is an important concern of this study and we intend to continue physical activity interventions after study is finished. Thus, employees of care facilities will be educated to guide the MEP. Furthermore, this approach ensures the opportunity for CG to participate in the MEP, which is an important ethical aspect.

### Challenges

There are several challenges in performing intervention studies in IWD. These are related to the selected study design as well as its target group and thus cannot be avoided. However, it is important to deal with these challenges to minimize their impact.

A big challenge in performing intervention studies with IWD is maintaining blinding to group allocation. Although all investigators will be blinded to group allocation, there is a potential risk that participants will disclose their group allocation during assessments. To minimize this risk, investigators will be asked not to talk about the intervention during assessments.

Working with IWD entails several general challenges as they are often suffering from frailty and multimorbidity. According to different motor and cognitive impairments in IWD, it is not possible to develop an intervention completely suitable for all participants. Hence, some adaptions of the intervention cannot be avoided. However, instructors are asked to minimize such adaptions and adhere to the manual as strictly as possible. Besides this, IWD are vulnerable in relation to attendance, adherence, and missing data. For instance, multiple motor and cognitive impairments partially prevent IWD from participating in all subassessments. Thus, attempts to enhance attendance and adherence as personal communication, support, or repeated invitation are planned.

Further challenges are seen in cooperation with care facilities. Employees assume important responsibilities, such as suggesting potential participants, assessing ADL and state of health, or supporting assessments and intervention. Restricted time or missing expertise is a potential risk for limitations. To reduce such limitations, employees will be provided detailed information on how to report required data and support for further problems.

### Implications and Perspectives

Findings of this study will be disseminated through publications and presentations (including information about important protocol modifications). Improving the defined primary outcomes is highly relevant considering the consequences of dementia-related motor deficits as stated in the introduction [35-37]. Insufficient amounts of physical activity also expedite existing motor and functional impairments in IWD [32,79]. Therefore, developing adequate physical activity interventions for IWD and offering guidelines is essential. We plan on publishing the MEP and communicating the underlying didactic concept of the training in a detailed manual if it proves to be effective.

This study will contribute to enhance scientific evidence and takes a first look at relations between motor and cognitive skills in IWD. The findings can also be directive for further investigations in the field of prevention, diagnosis, and therapy of dementia.

### Conclusions

There is a clear need for high-quality studies investigating the effectiveness of physical activity on motor and cognitive skills in IWD. Our study is mainly characterized by a dementia-specific intervention based on scientific knowledge, the combination of motor and cognitive tasks, and a large sample. Findings are highly relevant to influence the multiple motor and cognitive impairments of IWD often participating in limited physical activity. If the MEP proves to be effective, positive influences on everyday life are expected justifying its permanent implementation in care facilities.

### Multimedia Appendix

Multimedia Appendix 1. Sample session of the multimodal exercise program.

Multimedia Appendix 2. Description of the assessments.

## Appendix

Multimedia Appendix 1 Sample session of the multimodal exercise program.

Multimedia Appendix 2 Description of assessments.

## Abbreviations

ADAS-Cog: Alzheimer Disease Assessment Scale–Cognitive Subscale
ADL: activities of daily living
ANOVA: analysis of variance
CERAD: Consortium to Establish a Registry for Alzheimer's Disease
CONSORT: Consolidated Standards of Reporting Trials
CG: control group
E-ADL-Test: Erlangen Test of Activities of Daily Living
FICSIT-4: Frailty and Injuries: Cooperative Studies of Intervention Techniques 4
ICD-10: International Statistical Classification of Diseases and Related Health Problems, Tenth Edition
IG: intervention group
IWD: individuals with dementia
MEP: multimodal exercise program
MMSE: Mini-Mental State Examination
SPIRIT: Standard Protocol Items: Recommendations for Interventional Trials
