# Supplementary material for: Effectiveness of a 16-Week Multimodal Exercise Program on Individuals With Dementia: Study Protocol for a Multicenter Randomized Controlled Trial
Source: JMIR Res Protoc. 2017 Mar 3;6(3):e35. doi: 10.2196/resprot.6792 (PMC5357320; doi:10.2196/resprot.6792)
Supplement: Multimedia Appendix 1 [file resprot_v6i3e35_app1.pdf]

Multimedia Appendix 1 - Sample session

Table 1. Arrival of a sample session of the multimodal exercise program.

Imagination	Motor tasks	Cognitive tasks
Destination: ask the participants for destination of this session.	None.	If participants do not know destination, instructor gives explanations and descriptions about destination to create an imagination.
Pack your bag: take clothes out of the wardrobe.	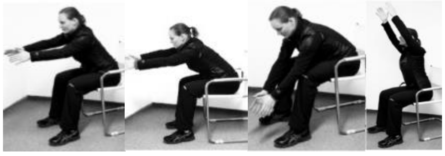<br>Move your arms and upper body with straightened back to the right, middle, and left side and then down to the floor, respectively (in order to put the clothes into the bag).	Instructor asks where in the wardrobe trousers (in middle compartment), T-shirts, and pullovers (in left overhead compartment), swimsuit or swim trunks (in right overhead compartment), etc. are.
Central Station: walk to the central station (meet the other tourists).	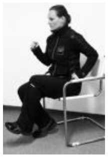<br>Alternately lift your legs and swing your arms to simulate walking.	None.
Greet your fellow passengers.	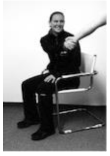<br>Upper body rotates to the left/right side and shake hands with fellow passenger.	None.
Take your ticket out of your pocket (trousers or shirt) or hand bag and show it to the conductor.	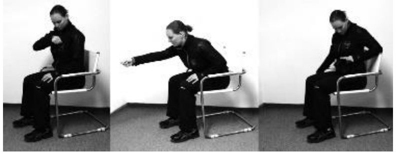<br>Bend down to touch the chair leg (handbag) (alternatively touch the addressed pocket of your clothes), then stretch 1 arm with straightened back to the front and hold it.	Participants should remember where they have put the ticket after the last journey.
The group reflects on packing and thinks about if everything important is included.	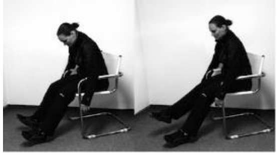<br>Resting and recovering.	Instructor asks participants what they packed in their bags a few minutes ago.
Get out of the train and walk to the hotel for check-in.	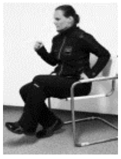<br>Alternately lift your legs and swing your arms to simulate walking.	None.

Table 2. Main part of a sample session of the multimodal exercise program (destination is a cruise in the Mediterranean Sea).

Imagination	Starting position	Motor tasks	Cognitive task	Time
**1st Holiday Event – Visiting Pisa**				
Leave the cruise ship, say goodbye to the captain.	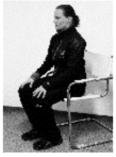<br>Sit up straight.	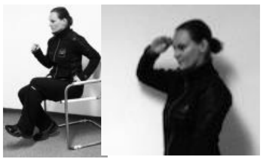<br>Alternately Lift your legs and wave to the captain and salute him with a tip on your forehead.	Instructor starts a conversation while simulating walking asking if participants have been in Italy/Pisa. If yes: “Can you tell us about your adventures?” If no: “What’s typical about Italy? Do you know some stereotypes?”	1min
Climb up the tower of Pisa.	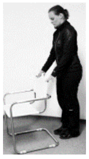<br>Stand behind the chair (hold on to the back of the chair).	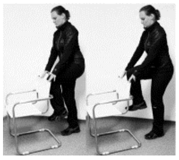<br>Alternately Lift your legs to your upper body while slightly leaning backwards/to the sides (due to the angle of the tower).	Participants count the stairs forward beginning with 1 (up to 15).	1min
Enjoy the amazing view.	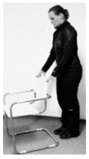<br>Stand behind the chair (hold on to the back of the chair).	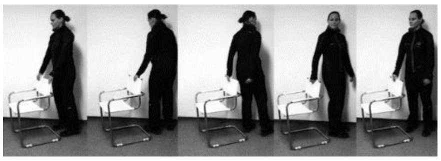<br>Turn around 360° (1 turn left, 1 turn right) (Keep holding on to the back of the chair if there is a risk of falling!)	None.	1x in each direction, 2min
Climb down the tower of Pisa.	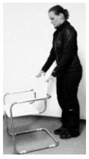	See above “climbing up”.	Participants count the stairs backwards beginning with 15.	1min
Return to the cruise ship and greet the captain.	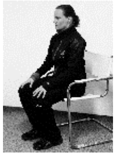		None.	1min

**2nd Holiday Event: Aqua Fitness in the deck pool of the cruise ship**				
Put on your swimsuit or swim trunks.	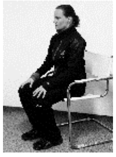 Sit up straight.	Lift your right leg and move your hands – starting at your upper leg – over your knee along your lower leg to your ankle and then up again until you reach your hip (simulating dressing up). Repeat the task on your other leg.	None.	2x each leg, 0.5min
Tasks with your pool noodle.	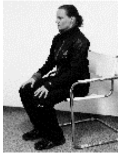 Sit up straight, pool-noodle in both hands.		None.	10min, 3 sets each task
		Hold pool-noodle with both hands (hands close to the ends). Stretch your arms vertically above your head. Put the pool-noodle behind your head – try to sit straight (it is allowed to bend the pool-noodle a little bit!).		3x
		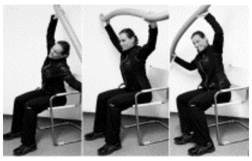 Hold pool-noodle with both hands (hands nearly at the ends). Stretch your arms vertically above your head. Lean your upper body to the right then to the left.		2x each side
		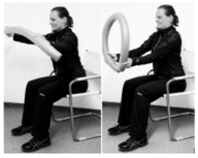 Horizontally hold the pool-noodle (hands close to the ends). Put both ends together to form a circle. Maximally extend your arms.		approx. 20sec
		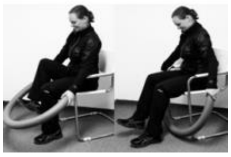 Bend the pool noodle in front of you (each end in 1 hand). Climb back and forth over the pool noodle (use it like a skipping rope).		3x back & forth

		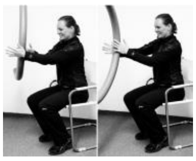 Vertically take the pool noodle between your legs, hold it in both hands with arms fully extended in front and roll it in your hands.		approx. 15sec
The Pool Bar invites your tourist group for a drink (5min break for drinking)				5min
**3rd Holiday Event: Arrive in Cairo**				
Leave the cruise ship, say goodbye to the captain and walk toward the camel station.	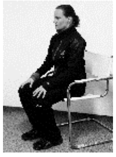	(See above “leave the cruise ship”).	None.	1min
Get up on the (sitting) camel. Due to the superstition of the camel drivers, you have to walk around the camel 3 times before sitting down.	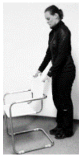 Stand behind the chair (hold on to the back of the chair).	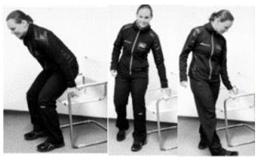 Walk 3 times around your chair, change direction each time you finished a round.	None.	4min
Ride the camel to the pyramids.	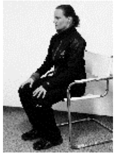 Sit up straight.	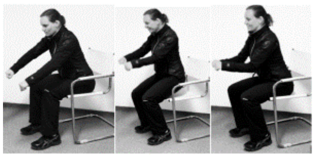 Take the reins → Arms are held in horizontal position while doing the other tasks. The camel moves slowly (wavers) → Hip tilts to the left/right/front/back while sitting on the chair. The camel gallops (hops) → rising a bit from the seat doing “ups” and “downs”.	Instructor tells the story about riding the camel above different barriers. At least 2 times participants have to ride slowly and gallop. Participants have to reply on the story by their movements.	2x 1min activity 0.5min pause, 1.5 min
Arrive at the pyramids and climb off the camel (same ritual like getting up on the camel).	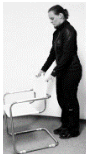	(See above “Get up on the (sitting) camel”).	None.	4min
Meet local Bedouins at the pyramids, participate in a traditional dance → repetitive dance choreography.	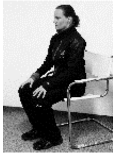 Sit up straight.	Common time (4 beats per cycle).	None.	10min

		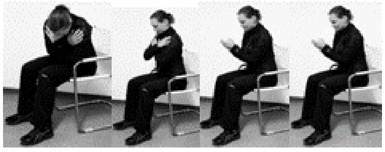<br>1st cycle: (1) put your right hand on the left shoulder and (2) the left hand on the right shoulder (→ arms crossed), (3) bow your upper body toward your right neighbor, then (4) sit straight again.		
		2nd cycle: (5) bow your upper body toward your right neighbor, then (6) sit straight again, (7 & 8) clap your hands twice.		
		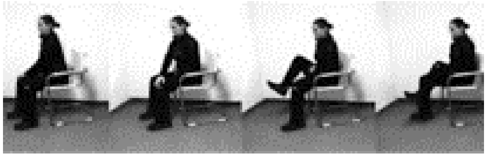<br>3rd cycle: (1) clap your left hand on your upper right leg, then (2) your right hand on your upper left leg, (3 & 4) tamp twice with your right foot.		
		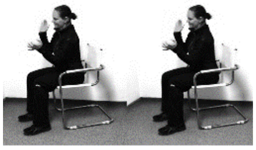<br>4th cycle: (5 & 6) stamp twice with your left foot, (7 & 8) clap your hands twice.		
Get up on the (sitting) camel.	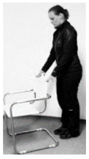	(See above "Get up on the (sitting) camel").	Participants should remember "The ritual".	4min
Ride back to the cruise ship.	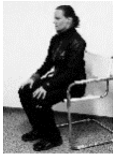	(See above "Ride the camel to the pyramids").	(Ssee above "telling a Story").	2x 1min activity 0.5min pause, 1.5min
Climb off the camel.	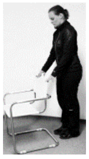	(See above "Get up on the (sitting) camel").	Together, instructor and participants verbally reconstruct the "ritual".	4min
Return to the cruise ship and greet the captain	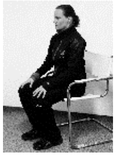	Return to the cruise ship and greet the captain.	None.	1min

Table 3. Departure of a sample session of the multimodal exercise program.

**Imagination**	**Motor tasks**	**Cognitive task**
Pack your bag.	None.	Participants have to remember the clothes and other things they have put in the bag on the arrival. Instructor encourages them by asking explicit questions.
Walk to the central station.	(See "Arrival").	None.
Take your ticket out of your pocket (trousers or shirt) or hand bag and show it to the conductor.	(See "Arrival").	Participants have to remember where they have left the ticket after they have showed it to the conductor.
Remembering.	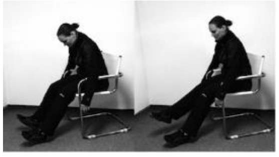 Relax and shake your arms and legs.	Participants have to remember what the destination of the journey today was and what experiences they had. Instructor encourages group to talk about the training lesson and if needed give hints.
Say goodbye to fellow passengers.	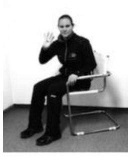 Wave your hands at other participants.	None.
Announcing the next destination of the upcoming training lesson.	None.	Instructor says goodbye to participants and give a brief outlook to the next training session.
