# Supplementary material for: Effectiveness of a 16-Week Multimodal Exercise Program on Individuals With Dementia: Study Protocol for a Multicenter Randomized Controlled Trial
Source: JMIR Res Protoc. 2017 Mar 3;6(3):e35. doi: 10.2196/resprot.6792 (PMC5357320; doi:10.2196/resprot.6792)
Supplement: Multimedia Appendix 2 [file resprot_v6i3e35_app2.pdf]

Multimedia Appendix 2 – Description of the assessments

Multimedia Appendix 2 gives a detailed description of performing each assessment. These are important information to attain a standardized testing procedure and ensure comparability.

FICSIT-4 Scale (static balance) [51]

Frailty and Injuries: Cooperative Studies of Intervention Techniques 4 scale (FICSIT-4) [51] determines static balance . Participants are asked to take different standing positions for 10 seconds each in the following order: Romberg, semi tandem, tandem, and single leg. First, the investigators demonstrate the positions and - if necessary - assist participants to take this position. Time is measured from the moment participants take up the position without help up to 10 seconds or in the following cases: foot position is changed or help is required to avoid a fall. Prerequisite for performing the next task of FICSIT-4 scale is the successful performance (10 seconds in the position) of the previous task. There is no test run before recording. The FICSIT-4 scale rates performance with 0 to 5 points according to number and time of finished positions [51].

Timed Up and Go test (mobility) [52]

Timed Up and Go test [52] assess mobility. For timed Up and Go test participants are asked to rise from a chair, walk 3 meters, turn around, go back, and sit down on the chair. Time recording starts with “Go” and stops when participants sit on the chair again. The chair has a sitting height of 46 centimeters and armrests. The distance of 3 meters is marked with a cone. Using a walking aid is allowed and is placed next to participants. Investigators demonstrate timed Up and Go test once and participants do one test run. During assessment, 3 instructions are allowed where needed: 1. “Go to the cone”, 2. “Turn around”, 3. “Sit down”. 2 valid trials are recorded.

6-meter walk test (mobility) [53]

The 6-meter walk test [53] assesses mobility and aims to capture normal gait speed. To reduce bias caused by the testing situation, participants are not explicitly informed about time keeping. A straight and flat distance of 6 meters is marked. During time keeping, investigators try to avoid conversation. All walking aids used in everyday life are applied. The 6-meter walk test is repeated 2 or 3 times if necessary.

GAITRite (gait parameters)

Temporal and spatial gait parameters will be analyzed using the electronic gait analysis system GAITRite (CIR Systems Inc, Franklin, NJ) with an active length of 4.88 meters, a spatial resolution of 1.27 centimeters, and a scan rate of 120 hertz. Gait parameters are recorded for 3 different conditions: walking with normal speed; walking with normal speed and the task of counting backwards from 50; walking with normal speed and the task of naming animals. All conditions will be repeated up to 5 times walking in the same direction to generate 3 valid trials. All walking aids used in everyday life are applied. To eliminate acceleration and deceleration during recording, participants start walking 2 meters in front of the GAITRite system and end 2 meters after [80]. Rests between trials are allowed when necessary.

Modified 30-second chair-stand test (strength of lower limbs) [54,55]

The modified 30-second chair-stand test [54,55] determines strength of lower limbs. Participants are asked to stand up and sit down as often as possible during 30 seconds. Repetitions are counted loudly. Moreover, the time to perform 5 repetitions is taken during the modified 30-second chair-stand test. In this modified version participants are allowed to use their arms [54,55]. The chair is the same as in timed Up and Go test (sitting height of 46 centimeters, with armrests). Investigators demonstrate the task and participants complete 1 test run. Valid performances, defined as hip angle during standing of about 180° and during

sitting of about 90°, are counted after the command “Go” with simultaneous timing up to 30 seconds. If 30 seconds end while standing, a semi repetition is counted. After a rest, fit participants complete a second trial without using arms with the same recording procedure as for the modified 30-second chair-stand test (including time for 5 repetitions).

Short physical performance battery (function of lower limbs) [56]

The short physical performance battery [56] evaluates function of lower limbs. It consists of standing balance (Romberg, semi tandem, tandem), gait speed, and 5 times sit to stand without using arms [56]. All measures are described above.

Erlangen Test of Activities of Daily Living (E-ADL-Test) (Activities of Daily Living) [59]

Erlangen Test of Activities of Daily Living (E-ADL-Test) [59] determines ADL. It consists of 5 items: pouring a drink, cutting a piece of bread, opening a small cupboard, washing hands, and tying a bow which will be performed during testing. A detailed description of each item is given by Graessel et al [59].

7-item physical performance test (Activities of Daily Living) [60]

The 7-item physical performance test [60] assess ADLs and includes the following tasks: writing a sentence, simulated eating, turning 360 degrees, putting on and removing a jacket, lifting a book and putting it on a shelf, picking up a penny from the floor, and a 50-foot walk test. The 7-item physical performance test will be performed according to the test protocol given by Reuben and Siu [60]. Due to time restrictions and to reduce physical stress, the 50-foot walk test will not be performed in this high-aged sample and the gait speed of the 6-meter walk test will be used instead.

Cognitive Assessments

All cognitive assessments will be performed and rated according to available test protocols.

Body mass and height

Body mass and height will be measured using a Seca 813 Robusta scale and Seca 213 stadiometer (Seca, Hamburg, Germany) with an accuracy of 0.1 kilogram and 0.1 centimeter, respectively. Participants will wear normal clothes and shoes during all measurements and the shoe type will be documented.

References

1. Berr C, Wancata J, Ritchie K. Prevalence of dementia in the elderly in Europe. European neuropsychopharmacology : the journal of the European College of Neuropsychopharmacology 2005;15(4):463-471 PMID:15955676
2. World Health Organization. Dementia: A Public Health Priority. Geneva, London: World Health Organization; Alzheimer's Disease International 2012 URL: https://extranet.who.int/agefriendlyworld/wp-content/uploads/2014/06/WHO-Dementia-English.pdf [accessed 2016-07-25]. Archived at: http://www.webcitation.org/6l3PInE1G.
3. Prince M, Guerchet M, Prina M. THE EPIDEMIOLOGY AND IMPACT OF DEMENTIA: CURRENT STATE AND FUTURE TRENDS 2015 URL: http://www.who.int/mental_health/neurology/dementia/dementia_thematicbrief_epidemiology.pdf [accessed 2016-07-25]. Archived at: http://www.webcitation.org/6mhjzrEvk.
4. Sosa-Ortiz AL, Acosta-Castillo I, Prince MJ. Epidemiology of dementias and Alzheimer's disease. Archives of medical research 2012;43(8):600-608 PMID:23159715
5. Prince M, Guerchet M, Prina M, Alzheimer's Disease International. Policy Brief: The Global Impact of Dementia 2013–2050 2013 URL: https://www.alz.co.uk/research/GlobalImpactDementia2013.pdf [accessed 2016-07-25]. Archived at: http://www.webcitation.org/6l3QmIoUc.

6. World Health Organization. The ICD-10 classification of mental and behavioural disorders: Clinical descriptions and diagnostic guidelines. Geneva 1992 URL: http://www.who.int/classifications/icd/en/bluebook.pdf?ua=1 [accessed 2016-07-25]. Archived at: http://www.webcitation.org/6l3QucFck.
7. Chew-Graham C, Ray M, editors. Mental health and older people: A guide for primary care practitioners. Switzerland: Springer; 2016. ISBN:978-3-319-29490-2.
8. Martyr A, Clare L. Executive function and activities of daily living in Alzheimer's disease: a correlational meta-analysis. Dement Geriatr Cogn Disord 2012;33(2-3):189-203 PMID:22572810
9. van Iersel MB, Hoefsloot W, Munneke M, Bloem BR, Olde Rikkert, M G M. Systematic review of quantitative clinical gait analysis in patients with dementia. Z Gerontol Geriatr 2004;37(1):27-32 PMID:14991293
10. Allan LM, Ballard CG, Burn DJ, Kenny RA. Prevalence and severity of gait disorders in Alzheimer's and non-Alzheimer's dementias. J Am Geriatr Soc 2005;53(10):1681-1687 PMID:16181166
11. Manckoundia P, Mourey F, Pfitzenmeyer P, Papaxanthis C. Comparison of motor strategies in sit-to-stand and back-to-sit motions between healthy and Alzheimer's disease elderly subjects. Neuroscience 2006;137(2):385-392 PMID:16289889
12. Groot C, Hooghiemstra AM, Raijmakers, P G H M, van Berckel, B N M, Scheltens P, Scherder EJA, van der Flier, W M, Ossenkoppele R. The effect of physical activity on cognitive function in patients with dementia: A meta-analysis of randomized control trials. Ageing Res Rev 2016;25:13-23 PMID:26607411

13. Angevaren M, Aufdemkampe G, Verhaar HJJ, Aleman A, Vanhees L. Physical activity and enhanced fitness to improve cognitive function in older people without known cognitive impairment. Cochrane Database Syst Rev 2008(3):CD005381 PMID:18646126

14. Hauer K, Schwenk M, Zieschang T, Essig M, Becker C, Oster P. Physical training improves motor performance in people with dementia: a randomized controlled trial. Journal of the American Geriatrics Society 2012;60(1):8-15 PMID:22211512

15. Kemoun G, Thibaud M, Roumagne N, Carette P, Albinet C, Toussaint L, Paccalin M, Dugue B. Effects of a physical training programme on cognitive function and walking efficiency in elderly persons with dementia. Dementia and geriatric cognitive disorders 2010;29(2):109-114 PMID:20150731

16. Rolland Y, Pillard F, Klapouszczak A, Reynish E, Thomas D, Andrieu S, Riviere D, Vellas B. Exercise program for nursing home residents with Alzheimer's disease: a 1-year randomized, controlled trial. Journal of the American Geriatrics Society 2007;55(2):158-165 PMID:17302650

17. Venturelli M, Scarsini R, Schena F. Six-month walking program changes cognitive and ADL performance in patients with Alzheimer. American journal of Alzheimer's disease and other dementias 2011;26(5):381-388 PMID:21852281

18. Christofoletti G, Oliani MM, Gobbi S, Stella F, Bucken Gobbi LT, Renato Canineu P. A controlled clinical trial on the effects of motor intervention on balance and cognition in institutionalized elderly patients with dementia. Clinical rehabilitation 2008;22(7):618-626 PMID:18586813

19. Brett L, Traynor V, Stapley P. Effects of Physical Exercise on Health and Well-Being of Individuals Living With a Dementia in Nursing Homes: A Systematic Review. J Am Med Dir Assoc 2016;17(2):104-116 PMID:26432622

20. Littbrand H, Stenvall M, Rosendahl E. Applicability and effects of physical exercise on physical and cognitive functions and activities of daily living among people with dementia: a systematic review. Am J Phys Med Rehabil 2011;90(6):495-518 PMID:21430516
21. Potter R, Ellard D, Rees K, Thorogood M. A systematic review of the effects of physical activity on physical functioning, quality of life and depression in older people with dementia. Int J Geriatr Psychiatry 2011;26(10):1000-1011 PMID:21905096
22. Blankevoort CG, van Heuvelen, Marieke J G, Boersma F, Luning H, Jong J de, Scherder EJA. Review of effects of physical activity on strength, balance, mobility and ADL performance in elderly subjects with dementia. Dement Geriatr Cogn Disord 2010;30(5):392-402 PMID:20980758
23. Suttanon P, Hill K, Said C, Dodd K. Can balance exercise programmes improve balance and related physical performance measures in people with dementia?: A systematic review. Eur Rev Aging Phys Act 2010;7(1):13-25 doi:10.1007/s11556-010-0055-8.
24. Pitkala K, Savikko N, Poysti M, Strandberg T, Laakkonen M-L. Efficacy of physical exercise intervention on mobility and physical functioning in older people with dementia: a systematic review. Exp Gerontol 2013;48(1):85-93 PMID:22960590
25. Heyn PC, Johnson KE, Kramer AF. Endurance and strength training outcomes on cognitively impaired and cognitively intact older adults: a meta-analysis. J Nutr Health Aging 2008;12(6):401-409 PMID:18548179
26. Forbes D, Forbes SC, Blake CM, Thiessen EJ, Forbes S. Exercise programs for people with dementia. Cochrane Database Syst Rev 2015(4):CD006489 PMID:25874613

27. Öhman H, Savikko N, Strandberg TE, Pitkala KH. Effect of physical exercise on cognitive performance in older adults with mild cognitive impairment or dementia: a systematic review. Dement Geriatr Cogn Disord 2014;38(5-6):347-365 PMID:25171577

28. Farina N, Rusted J, Tabet N. The effect of exercise interventions on cognitive outcome in Alzheimer's disease: a systematic review. Int Psychogeriatr 2014;26(1):9-18 PMID:23962667

29. Di Santo SG, Prinelli F, Adorni F, Caltagirone C, Musicco M. A meta-analysis of the efficacy of donepezil, rivastigmine, galantamine, and memantine in relation to severity of Alzheimer's disease. J Alzheimers Dis 2013;35(2):349-361 PMID:23411693

30. Matsunaga S, Kishi T, Iwata N. Combination therapy with cholinesterase inhibitors and memantine for Alzheimer's disease: a systematic review and meta-analysis. Int J Neuropsychopharmacol 2015;18(5) PMID:25548104

31. Matsunaga S, Kishi T, Iwata N. Memantine monotherapy for Alzheimer's disease: a systematic review and meta-analysis. PloS one 2015;10(4):e0123289 PMID:25860130

32. Hauer K, Becker C, Lindemann U, Beyer N. Effectiveness of physical training on motor performance and fall prevention in cognitively impaired older persons: a systematic review. Am J Phys Med Rehabil 2006;85(10):847-857 PMID:16998433

33. Huger D, Zieschang T, Schwenk M, Oster P, Becker C, Hauer K. Designing studies on the effectiveness of physical training in patients with cognitive impairment. Z Gerontol Geriatr 2009;42(1):11-19 PMID:18484197

34. Alexander NB, Hausdorff JM. Guest editorial: linking thinking, walking, and falling. The journals of gerontology. Series A, Biological sciences and medical sciences 2008;63(12):1325-1328 PMID: 19126844.

35. Guralnik JM, Ferrucci L, Simonsick EM, Salive ME, Wallace RB. Lower-extremity function in persons over the age of 70 years as a predictor of subsequent disability. N Engl J Med 1995;332(9):556-561 PMID:7838189

36. American Geriatrics Society, British Geriatrics Society, American Academy of Orthopaedic Surgeons Panel on Falls Prevention. Guideline for the Prevention of Falls in Older Persons. J Am Geriatr Soc 2001;49(5):664-672 PMID:11380764

37. Yumin ET, Simsek TT, Sertel M, Ozturk A, Yumin M. The effect of functional mobility and balance on health-related quality of life (HRQoL) among elderly people living at home and those living in nursing home. Arch Gerontol Geriatr 2011;52(3):e180-4 PMID:21167611

38. Vermeulen J, Neyens JCL, van Rossum E, Spreeuwenberg MD, Witte LP de. Predicting ADL disability in community-dwelling elderly people using physical frailty indicators: a systematic review. BMC Geriatr 2011;11:33 PMID:21722355

39. Telenius EW, Engedal K, Bergland A. Physical performance and quality of life of nursing-home residents with mild and moderate dementia. Int J Environ Res Public Health 2013;10(12):6672-6686 PMID:24317384

40. Chan A-W, Tetzlaff JM, Altman DG, Laupacis A, Gotzsche PC, Krleza-Jeric K, Hrobjartsson A, Mann H, Dickersin K, Berlin JA, Dore CJ, Parulekar WR, Summerskill WSM, Groves T, Schulz KF, Sox HC, Rockhold FW, Rennie D, Moher D. SPIRIT 2013 statement: defining standard protocol items for clinical trials. Ann Intern Med 2013;158(3):200-207 PMID:23295957

41. Chan A-W, Tetzlaff JM, Gotzsche PC, Altman DG, Mann H, Berlin JA, Dickersin K, Hrobjartsson A, Schulz KF, Parulekar WR, Krleza-Jeric K, Laupacis A, Moher D. SPIRIT 2013 explanation and elaboration: guidance for protocols of clinical trials. BMJ 2013;346:e7586 PMID:23303884

42. Schulz KF, Altman DG, Moher D. CONSORT 2010 statement: updated guidelines for reporting parallel group randomized trials. Ann Intern Med 2010;152(11):726-732 PMID:20335313

43. Moher D, Hopewell S, Schulz KF, Montori V, Gotzsche PC, Devereaux PJ, Elbourne D, Egger M, Altman DG. CONSORT 2010 explanation and elaboration: updated guidelines for reporting parallel group randomised trials. BMJ 2010;340:c869 PMID:20332511

44. Boutron I, Moher D, Altman DG, Schulz KF, Ravaud P. Extending the CONSORT statement to randomized trials of nonpharmacologic treatment: explanation and elaboration: Explanation and Elaboration. Ann Intern Med 2008;148(4):295-309 PMID:18283207

45. Reith W, Muhl-Benninghaus R. Differenzialdiagnose demenzieller Erkrankungen. [Differential diagnostics of dementia type diseases]. Der Radiologe 2015;55(5):378-385 PMID:25944275

46. Folstein MF, Folstein SE, McHugh PR. “Mini-mental state”. A practical method for grading the cognitive state of patients for the clinician. J Psychiatr Res 1975;12(3):189-198 PMID:1202204

47. Thurm F, Scharpf A, Liebermann N, Kolassa S, Elbert T, Lüchtenberg D, Woll A, Kolassa I-T. Improvement of Cognitive Function after Physical Movement Training in Institutionalized Very Frail Older Adults with Dementia. GeroPsych 2011;24(4):197-208 doi:10.1024/1662-9647/a000048.

48. Scharpf A, Servay S, Woll A. Auswirkungen von körperlicher Aktivität auf demenzielle Erkrankungen. Sportwiss 2013;43(3):166-180 doi:10.1007/s12662-013-0295-7.

49. Fabre C, Chamari K, Mucci P, Masse-Biron J, Prefaut C. Improvement of cognitive function by mental and/or individualized aerobic training in healthy elderly subjects. Int J Sports Med 2002;23(6):415-421 PMID:12215960

50. Ihl R, Weyer G. Alzheimer’s Disease Assessment Scale (ADAS) [Alzheimer Disease Assessment Scale (ADAS)]. Weinheim: Beltz Test 1993.

51. Rossiter-Fornoff JE, Wolf SL, Wolfson LI, Buchner DM. A cross-sectional validation study of the FICSIT common data base static balance measures. Frailty and Injuries: Cooperative Studies of Intervention Techniques. J Gerontol A Biol Sci Med Sci 1995;50(6):M291-7 PMID:7583799

52. Podsiadlo D, Richardson S. The timed “Up & Go”: a test of basic functional mobility for frail elderly persons: A Test of Basic Functional Mobility for Frail Elderly Persons. J Am Geriatr Soc 1991;39(2):142-148 PMID:1991946

53. Graham JE, Ostir GV, Kuo Y-F, Fisher SR, Ottenbacher KJ. Relationship between test methodology and mean velocity in timed walk tests: a review. Arch Phys Med Rehabil 2008;89(5):865-872 PMID:18452733

54. Jones CJ, Rikli RE, Beam WC. A 30-s chair-stand test as a measure of lower body strength in community-residing older adults. Res Q Exerc Sport 1999;70(2):113-119 PMID:10380242

55. Blankevoort CG, van Heuvelen, Marieke J G, Scherder EJA. Reliability of six physical performance tests in older people with dementia. Phys Ther 2013;93(1):69-78 PMID:22976448

56. Guralnik JM, Simonsick EM, Ferrucci L, Glynn RJ, Berkman LF, Blazer DG, Scherr PA, Wallace RB. A short physical performance battery assessing lower extremity function:

association with self-reported disability and prediction of mortality and nursing home admission. J Gerontol 1994;49(2):M85-94 PMID:8126356

57. Mahoney FI, BARTHEL DW. Functional evaluation: the Barthel index. Md State Med J 1965;14:61-65 PMID: 14258950

58. Lübke N, Meinck M, Renteln-Kruse W von. The Barthel Index in geriatrics. A context analysis for the Hamburg Classification Manual. [Der Barthel-Index in der Geriatrie. Eine Kontextanalyse zum Hamburger Einstufungsmanual]. Z Gerontol Geriatr 2004;37(4):316-326 PMID:15338161

59. Graessel E, Viegas R, Stemmer R, Kuchly B, Kornhuber J, Donath C. The Erlangen Test of Activities of Daily Living: first results on reliability and validity of a short performance test to measure fundamental activities of daily living in dementia patients. Int Psychogeriatr 2009;21(1):103-112 PMID:18925975

60. Reuben DB, Siu AL. An objective measure of physical function of elderly outpatients. The Physical Performance Test. J Am Geriatr Soc 1990;38(10):1105-1112 PMID:2229864

61. Reitan RM. Validity of the Trail Making Test as an Indicator of Organic Brain Damage. Percept Mot Skills 1958;8(3):271-276 doi:10.2466/pms.1958.8.3.271.

62. Reitan RM. Trail Making Test: Manual for administration and scoring 1992.

63. Elwood RW. The California Verbal Learning Test: Psychometric characteristics and clinical application. Neuropsychol Rev 1995;5(3):173-201 doi:10.1007/BF02214761.

64. Wilde NJ, Strauss E, Tulsky DS. Memory span on the Wechsler Scales. J Clin Exp Neuropsychol 2004;26(4):539-549 PMID:15512941

65. Shulman KI, Shedletsky R, Silver IL. The challenge of time: Clock-drawing and cognitive function in the elderly. Int J Geriatr Psychiatr 1986;1(2):135-140 doi:10.1002/gps.930010209.

66. Schwenk M, Zieschang T, Oster P, Hauer K. Dual-task performances can be improved in patients with dementia: a randomized controlled trial. Neurology 2010;74(24):1961-1968 PMID:20445152

67. Abernethy B. Dual-task methodology and motor skills research: Some applications and methodological constraints. Journal of Human Movement Studies 1988;14(3):101-132.

68. Luttenberger K, Schmiedeberg A, Grassel E. Activities of daily living in dementia: revalidation of the E-ADL Test and suggestions for further development. BMC psychiatry 2012;12:208 PMID:23176536

69. Morris JC, Mohs RC, Rogers H, Fillenbaum G, Heyman A. Consortium to establish a registry for Alzheimer’s disease (CERAD) clinical and neuropsychological assessment of Alzheimer’s disease. Psychopharmacol Bull 1988;24(4):641-652 PMID: 3249766

70. Morris JC, Heyman A, Mohs RC, Hughes JP, van Belle G, Fillenbaum G, Mellits ED, Clark C. The Consortium to Establish a Registry for Alzheimer’s Disease (CERAD). Part I. Clinical and neuropsychological assessment of Alzheimer’s disease. Neurology 1989;39(9):1159-1165 PMID:2771064

71. Lezak MD. Neuropsychological assessment. 5th ed. Oxford, New York: Oxford University Press; 2012. ISBN:0195395522.

72. LINN BS, LINN MW, GUREL LE. Cumulative illness rating scale. J Am Geriatr Soc 1968;16(5):622-626 doi:10.1111/j.1532-5415.1968.tb02103.x.

73. Faul F, Erdfelder E, Lang A-G, Buchner A. G*Power 3: a flexible statistical power analysis program for the social, behavioral, and biomedical sciences. Behav Res Methods 2007;39(2):175-191 PMID:17695343

74. Cohen J. Statistical Power Analysis for the Behavioral Sciences. 2 edition. Hillsdale, N.J.: Lawrence Erlbaum; 1988.

75. Saghaei M, Saghaei S. Implementation of an open-source customizable minimization program for allocation of patients to parallel groups in clinical trials. J Biomed Sci Eng 2011;4(11):734-739 doi:10.4236/jbise.2011.411090.

76. Cohen J. A Coefficient of Agreement for Nominal Scales. Educ Psychol Meas 1960;20(1):37-46 doi:10.1177/001316446002000104.

77. Shrout PE, Fleiss JL. Intraclass correlations: uses in assessing rater reliability. Psychol Bull 1979;86(2):420-428 PMID:18839484

78. Bossers WJR, van der Woude, Lucas H V, Boersma F, Scherder EJA, van Heuvelen, Marieke J G. Recommended measures for the assessment of cognitive and physical performance in older patients with dementia: a systematic review. Dement Geriatr Cogn Dis Extra 2012;2(1):589-609 PMID:23341825

79. Brach JS, FitzGerald S, Newman AB, Kelsey S, Kuller L, VanSwearingen JM, Kriska AM. Physical activity and functional status in community-dwelling older women: a 14-year prospective study. Arch Intern Med 2003;163(21):2565-2571 PMID:14638556

80. Kressig RW, Beauchet O. Guidelines for clinical applications of spatio-temporal gait analysis in older adults. Aging Clin Exp Res 2006;18(2):174-176 PMID:16702791
